# Revolutionizing SIEM Security: An Innovative Correlation Engine Design for Multi-Layered Attack Detection

**DOI:** 10.3390/s24154901

**Published:** 2024-07-28

**Authors:** Muhammad Sheeraz, Muhammad Hanif Durad, Muhammad Arsalan Paracha, Syed Muhammad Mohsin, Sadia Nishat Kazmi, Carsten Maple

**Affiliations:** 1 Department of Computer and Information Sciences, Pakistan Institute of Engineering and Applied Sciences, Islamabad 45650, Pakistan; msheeraz_18@pieas.edu.pk (M.S.); hanif@pieas.edu.pk (M.H.D.); arsalan_18@pieas.edu.pk (M.A.P.); 2 Department of Computer Science, COMSATS University Islamabad, Islamabad 45550, Pakistan; 3 College of Intellectual Novitiates (COIN), Virtual University of Pakistan, Lahore 55150, Pakistan; 4 Faculty of Automatic Control, Electronics and Computer Science, Silesian University of Technology, 44-100 Gliwice, Poland; sadia.nishat.kazmi@polsl.pl; 5 Cyber Security Centre, University of Warwick, Coventry CV4 7AL, UK

**Keywords:** SIEM, Industry 4.0, correlation engine, event filtering, OC, SEC, Hyperscan, DoS attack, MAC flooding attack, STP root take-over attack

## Abstract

Advances in connectivity, communication, computation, and algorithms are driving a revolution that will bring economic and social benefits through smart technologies of the Industry 4.0 era. At the same time, attackers are targeting this expanded cyberspace to exploit it. Therefore, many cyberattacks are reported each year at an increasing rate. Traditional security devices such as firewalls, intrusion detection systems (IDSs), intrusion prevention systems (IPSs), anti-viruses, and the like, often cannot detect sophisticated cyberattacks. The security information and event management (SIEM) system has proven to be a very effective security tool for detecting and mitigating such cyberattacks. A SIEM system provides a holistic view of the security status of a corporate network by analyzing log data from various network devices. The correlation engine is the most important module of the SIEM system. In this study, we propose the optimized correlator (OC), a novel correlation engine that replaces the traditional regex matching sub-module with a novel high-performance multiple regex matching library called “Hyperscan” for parallel log data scanning to improve the performance of the SIEM system. Log files of 102 MB, 256 MB, 512 MB, and 1024 MB, generated from log data received from various devices in the network, are input into the OC and simple event correlator (SEC) for applying correlation rules. The results indicate that OC is 21 times faster than SEC in real-time response and 2.5 times more efficient in execution time. Furthermore, OC can detect multi-layered attacks successfully.

## 1. Introduction

Digital transformation offers numerous benefits to organizations, making disconnection from the internet no longer an option [1] in the Industry 4.0 era. Industry 4.0, also known as the Fourth Industrial Revolution, refers to the contemporary trend of digitization, automation, and data exchange in manufacturing technologies encompassing a wide range of modern advancements, including Internet of Things (IoT), cyber-physical systems, cloud computing, artificial intelligence, augmented reality, and big data analytics for improved decision making [2,3]. The SIEM system provides a comprehensive suite of big data analytic capabilities aimed at optimizing decision-making processes.

Despite enormous benefits, connecting via the internet exposes an organization to a threat spectrum that spans the entire globe. The organization’s IT infrastructure must be protected with great care and vigilance. The organization must be aware of the security status of its network at all times to protect against malicious activity from inside or outside the organization. Recently, countless cyber threats have emerged and affected the world badly [4,5].

Cyberattacks, such as denial of service (DoS), distributed denial of service (DDoS), SQL injection (SQLi), cross-site scripting (XSS), cyber vandalism, espionage, web session hijacking, and ransomware attacks, have recently shaken up the world and led to severe data protection and privacy issues [4,5,6,7,8]. The beam-stealing attack [9], channel access attack [10], and more recent ransomware attacks have hit the world hard [11]. In 2020 alone, ransomware attacks were reasonably estimated to have paid out around USD 412 million [12]. Various security systems, such as firewalls, IDSs, IPSs, etc., have been deployed in organizations worldwide to protect IT infrastructures from such attacks. Data loss prevention (DLP) systems are also widely used to prevent data loss or leakage [13,14]. Various techniques, including machine learning-based methods, have been extensively used to improve the performance of such security systems [15,16]. Digital twin technology is also being used effectively in simulating various security attacks, ultimately aiding in the detection and prevention of such incidents [17].

Such security attacks and breaches steal and compromise sensitive information and disrupt routine operations [18,19]. They are also responsible for malfunctioning network devices, financial losses to the organization, and the spread of malware across the corporate network. Therefore, early detection and immediate prevention of such attacks are crucial [20]. At the same time, however, this task is becoming increasingly complex as such attacks have become very sophisticated. Furthermore, with the introduction of subnetting [21,22], supernetting [22], and virtual local area networks (VLANs) [23], today’s corporate networks have become very complex and complicated. Often, the latest malware goes undetected by individual security systems, pointing to the need for a layered approach to security.

The security devices work independently and, therefore, often lack the complete context of network traffic. This limits their ability to detect and prevent a sophisticated cyberattack. Contemporary sophisticated malware is quite advanced and often leaves no detectable traces when analyzed by security devices working independently. However, the chances of detecting the latest malware increase when log and contextual data from various devices in the network are analyzed collectively. Therefore, network and security devices deployed in the enterprise must work in coordination to detect and prevent such attacks.

The SIEM system is specifically designed for close coordination among various security devices [24]. The SIEM system works in coordination with other network and security devices in the network. The SIEM system provides a holistic view of corporate network security. The SIEM system receives logs from various devices in the corporate network, such as PCs, servers, switches, routers, firewalls, anti-virus programs, anti-malware, IDSs, IPSs, etc. These log files are analyzed by the SIEM system and the current security status of the corporate network is displayed. The SIEM system is located at a higher level than the other devices in the network. Therefore, the SIEM system is better for assessing the corporate network’s security status than the other security devices [25].

A SIEM system consists of various components that work together to achieve the overall functionality of the SIEM system. These components include log collection, normalization, correlation, storage, and visualization [26,27,28]. The correlation module (or engine) is the main (or core) component of an SIEM system [29]. The correlation engine analyzes various heterogeneous logs received from numerous log sources in the corporate network. The received log data are extensive and are not very meaningful due to their sizes. Using a correlation process, the correlation engine transforms the data into smaller and more meaningful chunks of data displayed via a graphical user interface (GUI) to show the current state of the organization’s security status [25]. The research community has proposed various techniques for the correlation process, including rule-based, finite-state machine-based, case-based reasoning, codebook-based, voting-based, explicit fault localization, dependency graphs, Bayesian networks, neural networks, etc. [30]. Moreover, artificial intelligence-based techniques and models have extensively been used for security event correlation [31].

In this study, we used a rule-based event correlation technique for the correlation engine. The correlation engine has rules that are applied to the injected log data. If a rule matches the input log data, an alarm is generated. As mentioned above, the SIEM system’s correlation engine transforms a large volume of less meaningful log data into a smaller, more coherent, and meaningful dataset. This correlation process includes the following steps:

1. Event filtering: Irrelevant log data are discarded to reduce the volume during the event filtering phase.
2. Event aggregation and de-duplication: The aggregation of closely related log data and merging of identical data occur in the event aggregation and de-duplication phase.
3. Event masking: Log data generated after a system failure is masked in this phase.
4. Root cause analysis: In the root cause analysis, dependencies between log data events are analyzed using tools such as dependency diagrams to explain log data events by other log data events.

When the SIEM system receives log data from various data sources within the corporate network, the volume of received data is immense. These sources include PCs, servers, switches, routers, IDS, IPS, firewalls, antivirus programs, web servers, DHCP servers, DNS servers, email servers, and more. An enterprise network may have hundreds or even thousands of such data sources. The log data arrive continuously and at high speeds, creating a massive amount of data that the SIEM system must receive, process, and store. In reality, the SIEM system’s correlation engine must process this vast amount of log data. Not only does the correlation engine need to extract a smaller, more meaningful portion of the data after analyzing the logs, but it also needs to complete this task as quickly as possible. The correlation process must be performed swiftly because if alerts are generated due to malicious activity on the network, immediate remedial action can be taken to protect the corporate network from further damage [25].

If the correlation engine does not process log data fast enough, this can have a detrimental effect on the entire corporate network. Log data can take too long to process, rendering remedial action pointless. This is because their effectiveness is severely limited when corrective actions are not taken promptly. Therefore, the main focus of this research work is to enhance the throughput performance of the conventional correlation engine of the SIEM system while improving the detection capabilities for multi-layered attacks. This will improve the ability of the SIEM system to detect malicious activities in the enterprise network, making the SIEM system more effective and useful [25]. The main contributions of this research work are as follows:

- In this research work, we propose and develop a novel correlation engine, the OC, which is a fast and efficient correlation engine using the high-performance multiple regex matching library “Hyperscan” for parallel log data scanning.
- Our proposed OC outperforms the traditional correlation engine significantly and improves the overall performance of the SIEM system while making it more effective and efficient in detecting cyberattacks.
- Our proposed OC has successfully detected some multi-layered attacks, including application layer and data-link layer attacks.

The rest of this paper is organized as follows. Section 2 analyzes the current state of the literature in this area. Section 3 presents the design of the proposed correlation engine and its implementation. Section 4 presents the experimental setup used in this research work. Section 5 deals with the results of the experiments and provides a detailed discussion of the obtained results. Section 6 tests the proposed correlation engine for detecting multi-layered attacks to verify its effectiveness. Section 7 discusses the correlation of logs originating from multiple log sources. Section 8 contains the conclusion of this research work.

## 2. Existing Approaches

The correlation engine is an integral component of all prevalent SIEM systems, such as ELK [32], IBM QRadar [33], Securoix [34], OSSIM [25], Exabeam [35], Splunk [36], LogRhythm [37], etc. The effectiveness of each of these SIEM systems is highly dependent on its correlation engine. Some SIEM systems, such as IBM QRadar, utilize proprietary correlation engines, while others, like OSSIM, employ rule-based correlation engines. Despite the extensive utilization of various pattern-matching algorithms and libraries by the correlation engines of these SIEM systems, it appears that the Hyperscan library has not been employed to the best of our knowledge.

There are several rule-based correlation engines for performing correlation tasks. These include SEC [38], Esper [39], Drools [40], NodeBrain [41], Prelude [42], OSSEC [43], OSSIM correlation engine [44], etc. Table 1 outlines the technical parameters of these correlation engines, including the development platform, memory requirements, platform dependency, and usability, which are used across various domains.

Various correlation engines are discussed and compared by the authors of [41]. The ‘Drools’ tool showed the best performance when the number of rules and input log events were high. By performance, we mean throughput and execution time. For better performance, throughput should be high while the execution time should be low. NodeBrain demonstrated optimal performance when the number of rules and input log events was low to medium. Esper demonstrated medium performance. SEC demonstrated mediocre performance, but its operational requirements, such as memory requirements, are low, making it particularly suitable for embedded systems and low-power devices. Apart from these performance parameters, some important factors, such as memory requirements, should also be considered. Drools failed to complete its execution in some scenarios due to its high memory requirement. So, considering all the factors, there is no specific correlation engine that is universally best suited for every situation.

The limitations and reasons for not using the correlation engines listed in the above table are given as follows:

- The Esper tool is primarily designed to work on a stream of events. It does not process text files rather it works on a stream of events coming into it.
- The Drools tool is not optimized for memory efficiency, as it struggled to complete some scenarios due to its high memory requirements.
- NodeBrain does not have file I/O capabilities; therefore, it cannot process text files.
- The Prelude correlator component is not independently available for use, rather, we have to install and configure the complete Prelude SIEM solution. In addition, we also need to understand PRL language for rule writing. More importantly, the Prelude correlator cannot directly process text files, rendering it infeasible for our integration scenario.
- The OSSEC tool can process text files, but the exact format of the stored data must be explicitly specified in its configuration file. In our integration scenario, we require the correlation engine to process text files with multiple data formats. Therefore OSSEC does not seem feasible in our integration scenario.
- The OSSIM correlation engine details are not available. Although it is free to use, it comes in a virtual machine and does not expose its correlation engine interface for use.

Among all of the correlation engines discussed above, SEC is the only correlation engine that meets our complete requirements. Its configuration and deployment are easy and intuitive as compared to the others. It is also platform-independent and requires less memory for its operation. This makes SEC a good choice for deployment in many environments. SEC is a lightweight correlation engine and runs as a single process. SEC can read input lines from a file, named pipe, or standard input and matches them against patterns that can be regular expressions or Perl subroutines. It correlates input events according to the rules defined in its configuration file. If an input event matches a rule, an alert is generated and stored in an output text file for further processing [45]. In [45], SEC is used for complex event processing in dynamic risk management architecture to enhance cyber situational awareness in organizations.

A monitoring architecture for large-scale HPC computing is presented in [46]. SEC and Splunk are used to detect certain conditions and then some responses are generated based on these conditions. Typically, such conditions are met by matching with regular expressions, and the responses are straightforward, such as generating a warning or marking a node as down. A framework for detecting anomalous messages from syslog log files was proposed [47]. In this framework, a rule-based correlation technique was used. Patterns are extracted from the syslog log files of the last few days or weeks, and these patterns are used to create SEC rules. The syslog log files are then compared with these rules. A message is classified as anomalous if it does not match these rules, and the messages that match the SEC rules are considered normal. The LogCluster algorithm is used to detect frequent message patterns in the syslog log files.

In [48], a tool metric visualization system (MVS) is presented. This tool has been set up for the dynamic visualization of network security events of a terrestrial trunked radio (TETRA) running in a software-defined networking (SDN) context. All network traffic going to a virtual machine is replicated by an SDN switch connected to it. Suricata IDS is used for intrusion detection. Suricata IDS rules related to VoIP are modified to monitor SIP over UDP network traffic. Rsyslog is used to transmit events to a central logging server. The SEC is used as a correlation engine in the SIEM system that analyzes selected events from the logs before sending them to the MVS for visualization.

In [49], the SEC correlation engine is discussed comprehensively, highlighting various important features alongside examples. Several rule set examples are provided to illustrate the capabilities of SEC. The exciting feature of integrating Perl code with SEC rules for custom netfilter event correlation is discussed in detail.

In [50], the SEC correlation engine was used for log-based distributed security event detection. The paper provides detailed insight into distributed security event detection. The centralized database-oriented log event correlation is quite common but it requires high network bandwidth, high system resources, and certain suspicious behaviors remain undetected. The research work uses SEC for quantitative evaluation with testing metrics that include bandwidth utilization, detection capability, and database query efficiency. The results reveal that the proposed architecture achieves a 99% reduction in network syslog traffic with a low false positive rate.

In [51], the Snort IDS and SEC correlation engine are integrated to reduce the number of alerts generated by Snort IDS. The MIT Defense Advanced Research Projects Agency (DARPA) dataset is used by Snort IDS to generate alerts, which are then correlated by SEC to filter and output only the most critical alerts for the security analyst. This removes the duplication caused by the same event coming from multiple IP addresses, significantly decreasing the number of events to be analyzed.

The SEC correlation engine has also been tested with SCADA systems. In [52], SEC is implemented in a SCADA system environment to detect system faults. The scenario discussed involves a mono-master system consisting of a local supervisor, one or more remote terminal units (RTUs), and a gateway for connection to the rest of the infrastructure. Numerous events generated by the SCADA system are stored in a log file, and corresponding correlation rules are created in the SEC rule file. SEC is applied to the log file, which contains log data from the SCADA system. By doing so, SEC successfully detects SCADA system faults, eliminating the need to manually scan through all the events in the log file.

In [53], a probabilistic approach is proposed for the detection of insider threats using log analysis and event correlation. The system architecture consists of four modules: log collection, log analysis, event correlation, and probability calculation. Log data are collected from multiple sources in the network and stored in a log file. The log file is then analyzed to remove redundant data. Next, the event correlation process is performed using SEC, and the probability of an insider threat is calculated at the end.

A useful and effective research paper was presented in [38]. Many new features that have recently been integrated into SEC were discussed. Best practices and recommendations to improve the performance of the SEC were listed, including the following:

- For better detection of attacks, several rules can be linked together in the correlation process. This provides improved context data for the correlation process.
- The support of Perl functions in SEC provides a useful platform for users to utilize and benefit from other Perl modules.
- The support of named match variables and the caching of matches provides a very effective feature for pattern matching and the use of Perl functions.
- Hierarchically organizing rule sets can enhance their effectiveness. When managing multiple rule sets and seeking to process data so that when one rule is triggered, unnecessary subsequent rules are bypassed, and the process jumps directly to a specified rule, the utilization of the “skip rules” keyword is exceptionally advantageous.

As can be seen from the above, SEC is used for correlation purposes in various settings due to its effectiveness. We also choose SEC for comparison with our proposed correlation engine for the following reasons:

- Rule-based.
- Ease of use.
- Fast learning curve.
- Low resource requirements.
- Good documentation and support.
- Simple deployment and configuration.
- Can read input from a file, named pipe, or standard input.

## 3. Proposed Correlation Engine Design

As mentioned earlier, the rule-based correlation engine performs numerous string comparisons or regex matches during its operation. So this is an important step in the overall operation of the correlation engine. If this step or process is slow, the performance of the correlation engine will be affected as this step is repeated very often. On the other hand, if this step or process is improved and becomes fast, the performance of the correlation engine increases significantly. This means that the performance of the SIEM system is improved and it becomes more effective and efficient. To improve the performance of the SIEM system through an efficient correlation engine, we have proposed and developed a novel correlation engine, the OC, in this study. In our proposed correlation engine, we transformed the slow and sequential regex matching operation of the correlation engine into a fast, efficient, and parallel pattern matching operation by replacing the traditional sequential regex matching process with a parallel multiple pattern matching process to improve the overall performance of the correlation engine and ultimately that of the SIEM system.

**Figure 1:**
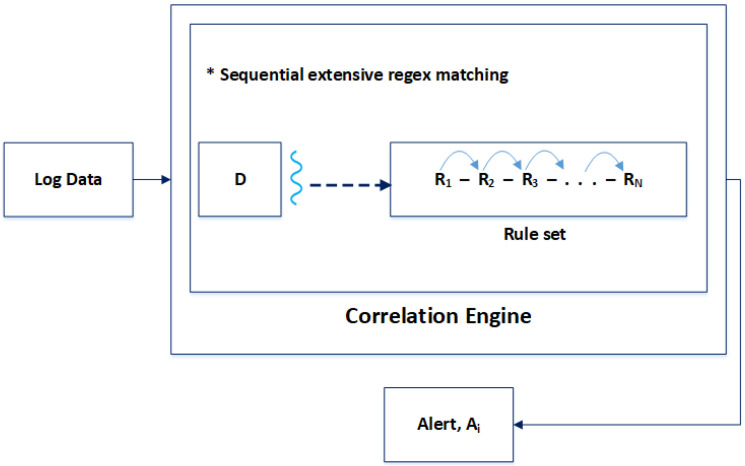
Traditional correlation engine.

Figure 1 illustrates how a traditional correlation engine, i.e., SEC works, where log data “D” are input into the correlation engine. The data “D” are sequentially matched against each rule “$R_{i}$” of the rule set. This process is tedious and time-consuming, generating an alert “$A_{i}$” whenever a match occurs. If the input log data “D” are large or the rule set contains numerous rules, more time will be required to complete this process, making this phase slow and impacting the performance of the correlation engine.

The above figure depicts the sequential nature of the correlation process. The first matter of concern is the log data “*D*”, which are not structured. If the log data are large, it is obvious that the correlation process will take quite some time to complete. Another concern regarding the correlation engine’s efficiency is the rule set’s sequential arrangement. The log data are matched sequentially against the rules, starting with “R_1_”, followed by “R_2_”, and so on. The other factor is one thread matching the entire log data against the rule set.

**Figure 2:**
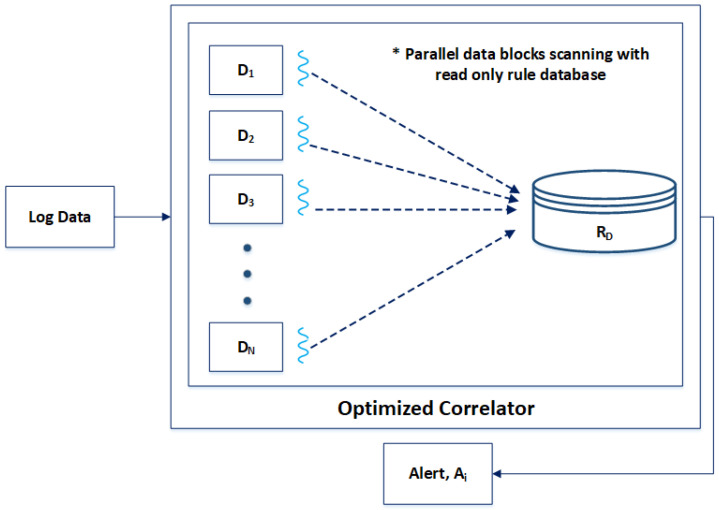
Proposed correlation engine.

Figure 2 shows the design of our proposed correlation engine, i.e., the OC. Our proposed correlation engine simultaneously processes multiple pattern matches, significantly speeding up the correlation process. In the proposed OC, when log data are input into the correlation engine, the log data are split into multiple data blocks “D*_i_*”. Each data block “D*_i_*” is picked up by a thread and is matched against the rules database “R*_D_*”. As much as the total number of threads supported by the system architecture can be created by the OC. The rules database “R*_D_*” is a read-only database, allowing for simultaneous access by multiple threads. This parallel processing of log data blocks is made possible through the “Hyperscan” library.

The performance difference between the traditional correlation engine, i.e., SEC, and our proposed OC is attributed to the aspects highlighted in Table 2.

**Figure 3:**
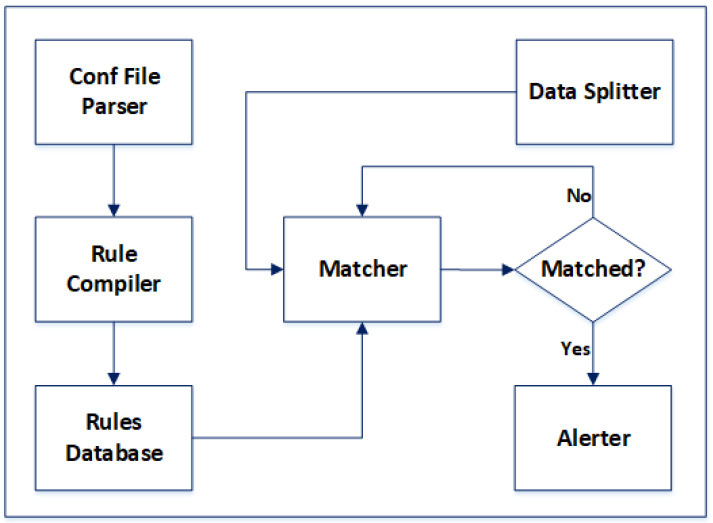
Block diagram of OC.

Table 2 highlights some of the key elements that contribute to the better throughput performance of the OC against the traditional correlation engine, i.e., SEC. The OC has been developed in C language, making it fast and efficient. It also has a multi-threaded architecture that enables it to exploit parallelism. Similarly, the SIMD architecture, multithreading support for rules database and data blocks, and use of the Hyperscan library are the key features that contribute to the better throughput performance of the OC compared to the SEC. Figure 3 shows the block diagram of the OC.

The Hyperscan library used in our proposed OC is an efficient and powerful platform for many regex pattern-matching applications, e.g., IDS/IPS [54]. The Hyperscan library, being open-source, allows for the simultaneous matching of a large number of regular expressions. Hyperscan provides a C application program interface (API) for various applications. The parallelism of the Hyperscan library makes the applications very fast. The Hyperscan library is a regular expression matching library that offers high performance, making it suitable for various security applications like firewalls, IDSs, IPSs, deep packet inspection (DPI) applications, etc. Algorithm 1 represents the working principle of the OC during its execution.
**Algorithm** **1:** Working principle of OC.
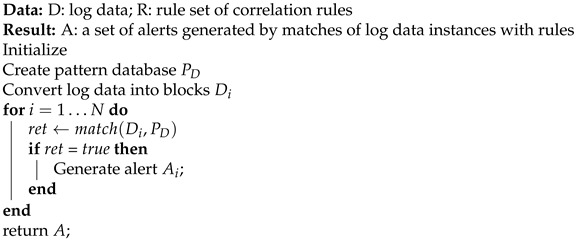


In the above algorithm, “*D*” represents the log data input into the OC, “*R*” represents the rule set of correlation rules, and “P*_D_*” represents the pattern database generated from the rule set. After initialization and creation of the pattern database, the input log data are converted into blocks, i.e., “D*_i_*”. Each log data block “D*_i_*” is matched against the pattern database “P*_D_*”, and if a match occurs an alert is generated, i.e., “A*_i_*”. At its core, Hyperscan utilizes two techniques [55,56], which are described below:

- Technique 1: Hyperscan uses a graph decomposition technique that converts regular expression matching into a series of string and finite automata matching. During this process, redundant operations are eliminated. The decomposed regular expressions increase the chances of a fast DFA match because they are smaller than the original pattern.
- Technique 2: Hyperscan also accelerates the matching of strings and finite automata by using single instruction, multiple data (SIMD) operations.

The general functionality of Hyperscan can be divided into two parts, namely, compile time and runtime, which are briefly described in Section 3.1 and Section 3.2, respectively.

### 3.1. Compile Time

**Figure 4:**
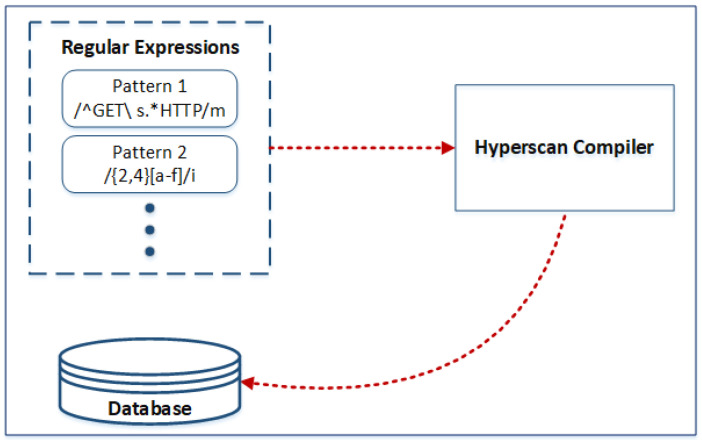
Hyperscan compile time.

The Hyperscan library contains a regular expression compiler written in C++. This compiler takes regular expressions as input and converts them into a pattern database through a complex process of graph analysis, decomposition, and optimization. This generated pattern database can be serialized and stored in memory for later use by the runtime. The compile-time part of Hyperscan is shown in Figure 4.

### 3.2. Run Time

**Figure 5:**
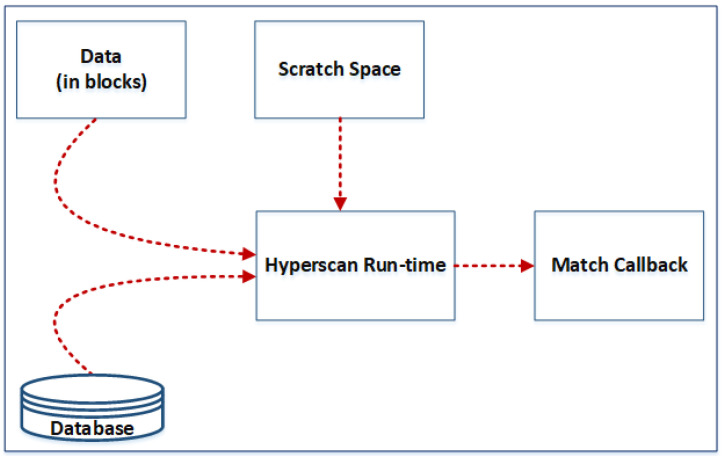
Hyperscan run time.

The Hyperscan runtime is shown in Figure 5 and has been developed in C++. The Hyperscan runtime requires a memory area called scratch space for its intermediate operations. This scratch space stores temporary information during the scanning process. The compiled pattern database is used to match the incoming data, which is divided into blocks. The internal Hyperscan matching engine uses deterministic finite automata (DFA) and non-deterministic finite automata (NFA) together with the Intel processor’s single instruction multiple data (SIMD) instructions to speed up the matching process. The user application receives the result of the matches via the callback function it provides. An important point here is that the compiled pattern database is read-only, which means that multiple CPU cores or threads can access this database simultaneously to increase the throughput of the matching process.

Using the Hyperscan library in the proposed OC instead of the traditional slow regex matching algorithm provides a performance boost due to the following key aspects.

- Hyperscan leverages algorithmic innovation by exploiting the Intel processor’s SIMD architecture, utilizing various SIMD registers to achieve superior performance in pattern-matching tasks, a capability lacking in traditional regex-matching algorithms.
- Hyperscan uses the multithreading approach, processing multiple pattern-matching tasks concurrently unlike the traditional regex-matching algorithm, giving it a performance boost.

The SIEM system used in this research work is proprietary and is called the cyber threat monitoring system (CTMS). In CTMS, logs from various PCs in the network are sent through the NXLog tool. The logs from switches, routers, and firewalls are sent through the syslog service. Once received and normalized by CTMS, these logs are temporarily stored in a text file. The OC is the correlation engine of the CTMS used for the rule-based correlation of logs. The OC processes this text file by applying rules that are present in its configuration file. Subsequently, CTMS stores these logs into the MySQL database.

## 4. Experimental Setup

**Figure 6:**
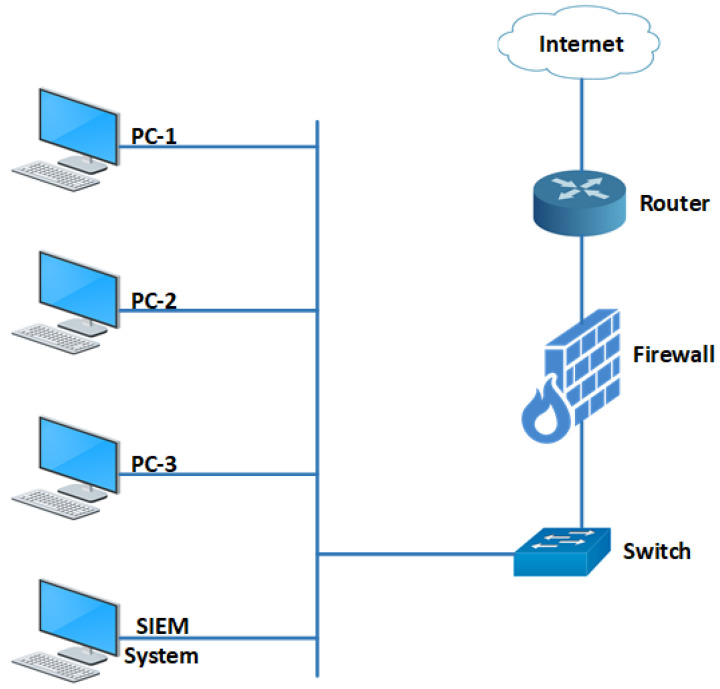
Experimental setup network diagram.

Our experimental network topology, shown in Figure 6, consists of several PCs and the SIEM system connected via a switch. The switch is connected to a firewall, which, in turn, is connected to the internet via a router. The SIEM system collects log data from various sources, including PCs, a switch, a firewall, and a router. NXLog, a free log collection tool, receives log data from various sources. NXLog is used for the log collection mechanism as it supports Windows, Linux, macOS, and Android operating systems. The architecture of the NXLog tool is agent-based. Agents are installed on all PCs in the network that send log data to the NXLog server installed on the SIEM system [57]. We used PCs with both Windows and Linux operating systems to obtain log data from different operating systems.

The SIEM system includes our proposed OC and SEC applications. Once we have collected a reasonable amount of data, we apply SEC and the proposed OC to the collected log files of different sizes. The main difference between these two correlation engines is that SEC uses the standard pattern-matching mechanism of Perl, while our proposed OC uses Hyperscan for pattern matching. The simulation results are compared based on real-time response and system time/execution time. Real-time response is the time an application starts its execution and lasts until its completion. The system time/execution time is the time it takes for an application to be executed by the CPU. SEC and OC are executed on a virtual machine. The details of the system and the virtual machine on which SEC and OC are executed are listed in Table 3.

### 4.1. Assumptions

In the above experimental setup, it is assumed that

- The PC-1 and PC-2 machines run the Windows operating system (Windows 10), while the PC-3 machine operates on the Linux operating system (CentOS-7). The SIEM system machine operates on the CentOS-7 operating system, and the SIEM software, which we call the Cyber Threat Monitoring System (CTMS-ver 1.0, CIPMA Lab, PIEAS, Islamabad, Pakistan), is installed on it.
- The logs collected at the SIEM system through the NXLog tool should contain logs for failed login attempts into three PC machines along with many other OS logs.
- The rule file contains two rules for detecting failed login attempts for Windows and Linux operating systems.
- The SIEM System machine has an Intel processor with SIMD architecture to be used by the OC’s Hyperscan module.

### 4.2. Limitations

One limitation of the proposed architecture is its dependency on Intel processors. Consequently, our proposed OC can only run on an Intel processor offering SIMD architecture that is needed by the Hyperscan module of the OC.

## 5. Results and Discussion

**Figure 7:**
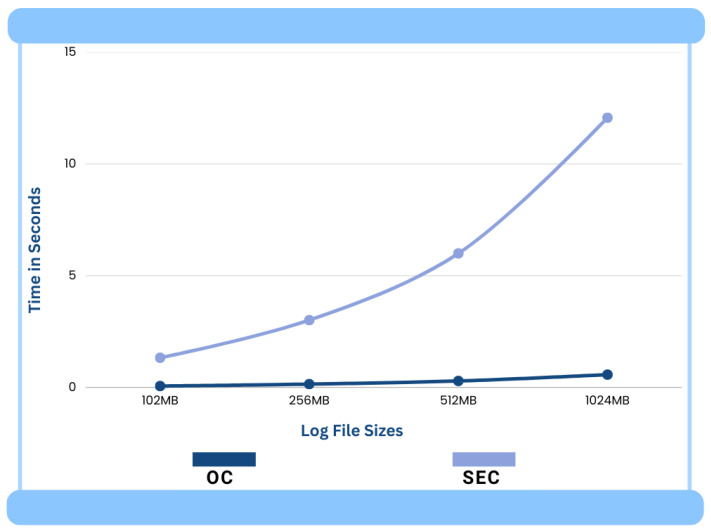
Average real times.

**Figure 8:**
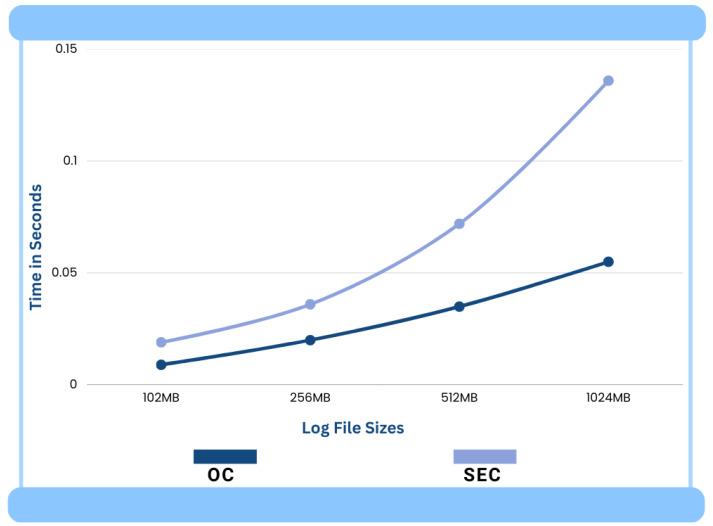
Average system times.

The log files of different sizes were selected for the experiment to understand the performance of the correlation engines OC and SEC. The log files of sizes 102 MB, 256 MB, 512 MB, and 1024 MB were input into the OC and SEC. Only two rules were included in the rule file as we only wanted to check the performance of the two correlation engines in pattern recognition. The OC and SEC were run 5 times to process each log data file to obtain a fair performance comparison. The average real-time and system-time statistics for both correlation engines (OC and SEC) for different sizes of log files can be seen in Figure 7 and Figure 8, respectively.

Figure 7 shows the difference in the performance of the correlation engines of OC and SEC in terms of the average real-time response and our proposed OC shows about 21 times better performance than the SEC. This is a significant performance improvement. This improved performance of OC is mainly due to the fast Hyperscan pattern-matching technique. This performance becomes even more apparent and obvious when OC is run in real-time with continuously arriving logs.

Figure 8 shows the performance comparison between OC and SEC for application system times/execution times. Also, in this case, OC shows a performance that is about 2.5 times better than SEC. This better performance is again attributed to Hyperscan’s fast and efficient pattern-matching technique.

The preceding discussion indicates that incorporating OC into the SIEM system has the potential to enhance its performance. However, understanding the integration of OC with the SIEM system is essential. In our implementation, logs from various sources are temporarily stored in a text file before being saved in the database. The OC processes this text file by applying pre-fed rules to generate output if any rule is matched. Consequently, integrating and deploying OC within the existing SIEM system architecture may present a potential challenge, as OC needs a text log file as input for its operation. The OC is also quite scalable in terms of handling the amount of data. When OC is applied to the text file, it processes the data and then clears the file, allowing for additional data to be appended.

## 6. Detection of Multi-Layered Attacks Using OC

We use the same experimental setup as in Figure 6 to verify and validate the effectiveness of our proposed OC for multi-layered attacks. We selected the application layer and data-link layer attacks, including denial-of-service (DoS) attacks, file transfer protocol (FTP) login attacks, system login attacks, switch media access control (MAC) flooding attacks, and switch spanning tree protocol (STP) take-over attacks.

### 6.1. Detection of DoS Attack

The denial-of-service (DoS) attack targets the OSI application layer and exploits design or implementation vulnerabilities in an algorithm [58]. The main goal of a DoS attack is to disrupt the service provided by an application. We launch the DoS attack from the PC-1 machine on a Windows 7 virtual machine (VM) running on the PC-2 machine. A Kali Linux VM is running on the PC-1 machine. The Windows 7 VM hosts an Apache server-based application accessible via its IP address. We launch four types of DoS attacks on the Apache server using tools provided by the Kali Linux operating system, including simple golden eye, random golden eye, slow HTTP, and slow Loris DoS attacks. These DoS attacks are executed using the following commands:

- Simple GoldenEye DoS attack:
  ./goldeneye.py http://victim-machine-ip:80 -s 1000
- Random GoldenEye DoS attack:
  ./goldeneye.py http://victim-machine-ip:80 -w 10 -s 1000 -m random
- Slow HTTP DoS attack:
  slowhttptest -c 500 -H -g -o ./output-file -i 10 -r 200 -t GET -u http://victim-machine-ip -x 24 -p 2
- SlowLoris DoS attack:
  slowloris victim-machine-ip -s 500

During the execution of the DoS attacks, the application hosted on the Apache server becomes inaccessible. This occurs because the DoS attacks exhaust all resources of the Apache server, rendering it unable to process further requests. The same type of Apache server error log is generated for all four types of DoS attacks. This uniformity is advantageous as it enables the creation of a generic correlation rule capable of detecting all these DoS attacks. The error log generated by the Apache server for these DoS attacks is shown below.

[Mon Apr 03 15:39:23.582048] [mpm_winnt:error] [pid 1196:tid 4020] AH00326: Server ran out of threads to serve requests. Consider raising the ThreadsPerChild setting

Although the error logs for all these attacks are identical, the access logs vary slightly. After analyzing these Apache web server logs, we created the DoS attack detection rule, illustrated below. The rule is a single-type rule, using a regular expression pattern type. It examines the specified log and matches it against the expression defined in the rule.

type=Single
ptype=RegExp
pattern=\[pid \d+\:tid \d+] \S+\d+\: Server ran out of threads to serve requests. Consider raising the ThreadsPerChild setting
desc=DoS attack
action=write alert_file Alert - DoS attack detected.

### 6.2. Detection of FTP Attack

We launched an FTP login attack, an application layer attack, from the PC-1 machine to the PC-2 machine. The PC-1 machine runs a Kali Linux VM, and the PC-2 machine hosts the FTP service. We used a tool provided by the Kali Linux VM to execute a brute-force attack on the FTP login with the following command:
ftp_login host=victim-machine-ip user=FILE0 password=FILE1 0=usernames.txt 1=passwordlist.txt -x ignore:mesg=‘Login incorrect.’


The Kali Linux tool attempted to use numerous usernames and passwords for FTP login. Many of these combinations were rejected as invalid by the FTP service running on the PC-2 machine. When an invalid username and password combination was used for FTP login, a log entry was recorded in the log file of the FTP service, specifically in the vsftpd.log file. An example log entry for an invalid FTP login attempt is shown below.
Mon Apr 3 17:06:15 2023 [pid 3502] [ubuntu] FAIL LOGIN: Client “::ffff:192.168.99.160”


The log of a failed FTP login attempt includes a timestamp, process ID, message, and the system IP address that attempted the FTP login. After thoroughly analyzing the log, we created a rule for detecting a failed FTP login attempt, shown below.

type=Single ptype=RegExp pattern=(\S+ \S+ \d+ \d+\:\d+\:\d+ \d+) \[pid\d+\] \[\S+\] FAIL LOGIN\: Client \"\:\:\S+\:(\d+\.\d+\.\d+\.\d+)\" desc=Failed FTP login attempt action=write alert_file A failed FTP login attempt detected from $2 to $1

### 6.3. Detection of Failed Login Attack

We launched an SSH/login attack—an application layer attack—from the PC-1 machine to the PC-2 machine, which hosts a Windows 7 VM. When attempting to log in to the Windows 7 VM with an incorrect password, the operating system records a failed login attempt. An example log entry for a failed login attempt in the Windows 7 operating system is shown below.
{"EventTime":"2024-06-16 15:22:34","Hostname":"CTMS-Client","Keywords":-921886 8437227405312,"EventType":"AUDIT_FAILURE","SeverityValue":4,"Severity": "ERROR","EventID":4625,"SourceName":"Microsoft-Windows-Security-Auditing", "ProviderGuid":"54849625-5478-4994-A5BA-3E3B0328C30D","Version":0,"Task":12544, "OpcodeValue":0,"RecordNumber":71996,"ActivityID":"20804495-9F3D-0004-E244- 80203D9FD901","ProcessID":1060,"ThreadID":14464,"Channel":"Security","Message": "An account failed to log on.\r\n\r\nSubject:\r\n\tSecurity ID:\t\tS-1-5- 18\r\n\tAccount Name:\t\tCTMS-CLIENT$\r\n\tAccount Domain:\t\t WORKGROUP\r\n\tLogon ID:\t\t0x3E7\r\n\r\nLogon Type:\t\t\t2\r\n\r\n Account For Which Logon Failed:\r\n\tSecurity ID:\t\tS-1-0-0\r\n\tAccount Name:\t\tclient\r\n\tAccount Domain:\t\tCTMS-CLIENT\r\n\r\n\tFailure Reason:\t\tUnknown user name or bad password.\r\n\t. . . }

The log entry for a failed login attempt includes several critical fields such as EventTime, Hostname, SeverityValue, Severity, SourceName, Message, SourceIP, Platform, etc. Among these, the “Message” field is particularly crucial as it identifies the failed login attempt of a user. We developed an effective rule for detecting failed login attempts, illustrated below.

type=Single ptype=RegExp pattern=\{\"EventTime\"\:\"(\d+\-\d+\-\d+ \d+\:\d+\:\d+)\"\,\"Hostname\"\: \"(\S+)\"\,\"Keywords\"\:\-\d+\,\"EventType\"\:\"\S+\"\,\"SeverityValue\"\: \d+\,\"Severity\"\:\"\S+\"\,\"EventID\"\:\d+\,\"SourceName\"\:\"\S+\"\, \"ProviderGuid\"\:\"\\d+\-\d+\-\d+\-\S+\-\S+\\"\,\"Version\"\:\d+\, \"Task\"\:\d+\,\"OpcodeValue\"\:\d+\,\"RecordNumber\"\:\d+\,\"ActivityID \"\:\"\\d+\-\S+\-\d+\-\S+\-\S+\\"\,\"ProcessID\"\:\d+\,\"ThreadID\"\:\d+\, \"Channel\"\:\"\S+\"\,\"Message\"\:\"\S+ \S+ \S+ \S+ \S+ \S+ desc=An account failed to log on action=write alert_file NIL * 2∗1 * Failed login attempt detected on $2 machine at $1

### 6.4. Detection of MAC Flooding Attack

The MAC flooding attack targets the second OSI layer, i.e., the data-link layer. In this attack, the attacker floods the switch with numerous spoofed MAC addresses to consume its limited memory for the MAC address table. Consequently, the switch behaves like a network hub, sending frames to all ports. An attacker connected to one of the switch ports can then intercept all frames [59]. We launch a MAC flooding attack from the PC-1 machine to the Dell switch. When the PC-1 machine, connected to port 18 of the switch, begins flooding MAC addresses, the switch saves some log entries. A log entry for a MAC flooding attack is given below.

{"MessageSourceAddress":"192.168.99.2","EventReceivedTime":"2023-07-12 12:18:12", "SourceModuleName":"udp_two","SourceModuleType":"im_udp", "SyslogFacilityValue":23,"SyslogFacility":"LOCAL7","SyslogSeverityValue":5, "SyslogSeverity":"NOTICE","SeverityValue":2,"Severity":"INFO","Hostname": "DellN2048-1","EventTime":"2023-07-12 12:18:44","SourceName":"TRAPMGR", "ProcessID":"dtlAddrTask","Message":"traputil.c(763) 2001489 %% MAC Lock Violation: Gi1/0/18 , 3c:2c:30:c2:ce:b9, vlan 1","Platform":"Switch"}

These log entries are then sent to the SIEM system, where the correlation engine processes them using the pre-defined rule, successfully detecting the MAC flooding attack, as shown below.

type=Single ptype=RegExp pattern=\{\"MessageSourceAddress\"\:\"(\d+\.\d+\.\d+\.\d+)\"\, \"EventReceivedTime\"\:\"(\d+\-\d+\-\d+ \d+\:\d+\:\d+)\"\, \"SourceModuleName\"\:\"\S+\"\,\"SourceModuleType\"\:\"\S+\"\, \"SyslogFacilityValue\"\:\d+\,\"SyslogFacility\"\:\"\S+\"\, \"SyslogSeverityValue\"\:\d+\,\"SyslogSeverity\"\:\"\S+\"\,\"SeverityValue\ "\:\d+\,\"Severity\"\:\"\S+\"\,\"Hostname\"\:\"\S+\"\,\"EventTime\"\: \"\d+\-\d+\-\d+ \d+\:\d+\:\d+\"\,\"SourceName\"\:\"\S+\"\,\"ProcessID\"\: \"\S+\"\,\"Message\"\:\"\S+ \d+ %% MAC Lock Violation\: \S+ \, \S+\:\S+\:\S+\:\S+\:\S+\:\S+\, \S+ \d+\"\,\"Platform\"\:\"\S+\"\} desc=Detection of Switch MAC flooding attack action=write alert_file MAC flooding attack detected on $2 machine at $1

### 6.5. Detection of STP Root Take-Over Attack

The spanning tree protocol (STP) is a LAN-based protocol to prevent loops in the network topology. However, STP has vulnerabilities that attackers often exploit. In an STP root takeover attack, an attacker attempts to change or take over the root bridge in the network to disrupt its functionality [60]. This attack targets the data-link layer (OSI layer 2), and we launch it from the PC-1 machine on the network switch. The Dell switch in the network has port security enabled, preventing the attacker from modifying or taking over the root switch, but the switch recognizes this attack. The log generated by this attack on the switch is shown below.

{"MessageSourceAddress":"192.168.99.2","EventReceivedTime":"2023-07-12 14:48:39" , "SourceModuleName":"udp_two","SourceModuleType":"im_udp", "SyslogFacilityValue":23,"SyslogFacility":"LOCAL7","SyslogSeverityValue":3, "SyslogSeverity":"ERR","SeverityValue":4,"Severity":"ERROR","Hostname": "DellN2048-1","EventTime":"2023-07-12 14:49:11","SourceName":"DOT1S", "ProcessID":"dtlTask","Message":"dot1s_txrx.c(269) 9033062 %% dot1sBpduReceive(): Discarding the BPDU, since it is an invalid BPDU type","Platform":"Switch"}

This log entry is then forwarded to the SIEM system, where the correlation engine processes it using the pre-configured rule, as shown below. Our proposed correlation engine, the OC, successfully detects this attack.

type=Single ptype=RegExp pattern=\{\"MessageSourceAddress\"\:\"(\d+\.\d+\.\d+\.\d+)\"\, \"EventReceivedTime\"\:\"(\d+\-\d+\-\d+ \d+\:\d+\:\d+)\"\, \"SourceModuleName\"\:\"\S+\"\,\"SourceModuleType\"\:\"\S+\"\, \"SyslogFacilityValue\"\:\d+\,\"SyslogFacility\"\:\"\S+\"\, \"SyslogSeverityValue\"\:\d+\,\"SyslogSeverity\"\:\"\S+\"\,\"SeverityValue\"\: \d+\,\"Severity\"\:\"\S+\"\,\"Hostname\"\:\"\S+\"\,\"EventTime\"\:\"\d+\- \d+\-\d+ \d+\:\d+\:\d+\"\,\"SourceName\"\:\"\S+\"\,\"ProcessID\"\:\"\S+ \"\,\"Message\"\:\"\S+ \d+ %% dot1sBpduReceive\(\)\: Discarding the BPDU\, since it is an invalid BPDU type\"\,\"Platform\"\:\"\S+\"\} desc=Detection of Switch STP Root change attack action=write alert_file STP Root change attack detected on $1 machine at $2

Our proposed correlation engine, the OC, successfully detected multi-layered attacks, including DoS, FTP login, system login, MAC flooding, and STP root takeover attacks. This demonstrates that OC can be further tuned to detect other types of attacks by adding appropriate correlation rules for those attacks. A summary of the detection of multi-layered attacks is presented in Table 4.

## 7. Correlating Multiple Devices Logs

**Figure 9:**
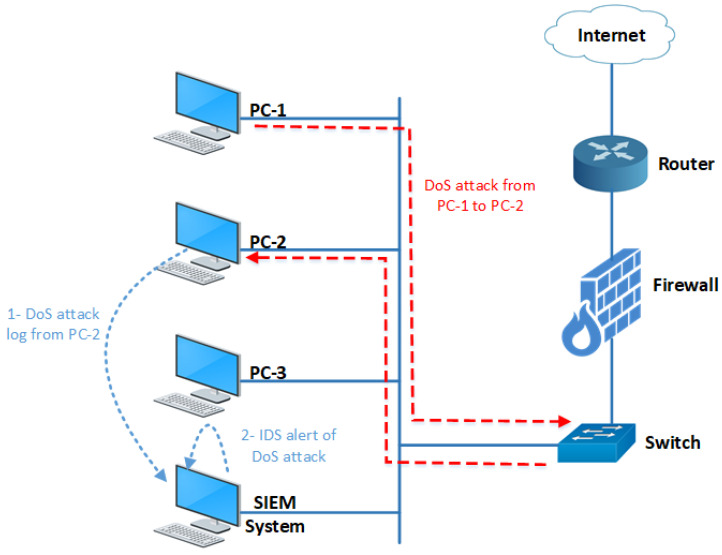
DoS attack correlation process.

All the above attacks are detected by analyzing logs from a single device. However, the correlation engine can correlate logs from multiple devices on the network. In some scenarios, an attack is detected by analyzing and correlating logs from multiple devices. Figure 9 illustrates a network scenario where a DoS attack is launched and detected by correlating logs from multiple sources.

In this scenario, a DoS attack is launched from the PC-1 machine to the PC-2 machine, with both computers connected via the same switch. This configuration results in the generation of DoS attack logs on two source devices.

- First, the DoS attack traffic, after being generated by the PC-1 machine, is routed through the switch and reaches the PC-2 target machine. The PC-2 machine, running an Apache server-based application vulnerable to DoS attacks, generates logs related to this DoS attack traffic. Consequently, logs related to the DoS attack are generated on the victim machine, as shown below.
[Mon Apr 13 10:15:33.582048 2023] [mpm_winnt:error] [pid 1196:tid 4020] AH00326: Server ran out of threads to serve requests. Consider raising the ThreadPerChild setting
- Second, mirror traffic is forwarded from a switch port to the SIEM system machine running Snort IDS. Snort IDS detects the DoS attack originating from the PC-1 machine to the PC-2 machine using predefined rules, generating an alert or log, as shown below. This alert is then sent to the SIEM system for analysis and correlation by the correlation engine. 
04/13-10:15:33.584030 [**] [1:1000001:1] DoS attack detected. [**] [Classification: DoS attack event] [Priority: 3] TCP 192.168.99.10 -> 192.168.99.20

After obtaining DoS attack logs from the PC-2 machine and the IDS, a rule is written to correlate these two logs, which enables the correlation engine (OC) to detect the DoS attack as shown below.


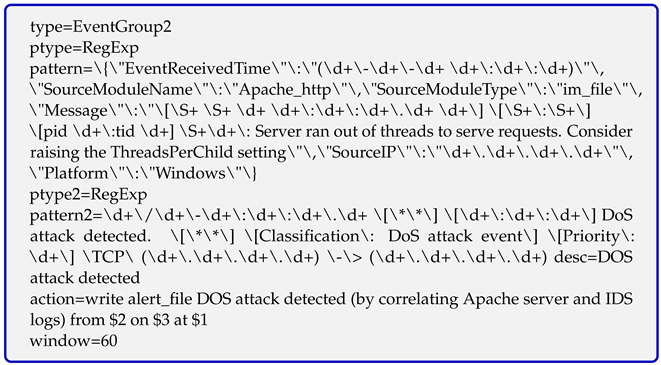


The simulation results demonstrate that our correlation engine (OC) successfully detects DoS attacks by correlating the logs of the PC-2 machine and the IDS system using a correlation rule. Similarly, more correlation rules can be added to detect a wider range of cyberattacks by correlating logs from multiple log sources.

## 8. Conclusions and Future Work

A SIEM system has become indispensable for security management for companies in the age of Industry 4.0. The correlation engine is the core module of the SIEM system. The performance of the correlation engine has a great impact on the overall performance of the SIEM system. The correlation engine performs a large number of pattern-matching tasks during its execution. Therefore, this step must be efficient and fast to improve the performance of the SIEM system. In this research work, we proposed a fast and efficient correlation engine, i.e., the OC. The OC uses the “Hyperscan” library at its core for parallel log data scanning. The log data received from various devices in the network are stored in log data files of 102 MB, 256 MB, 512 MB, and 1024 MB. The log data files are input into the OC and SEC for applying correlation rules. The findings indicate that OC outperforms SEC significantly, being 21 times more efficient in real-time response and 2.5 times faster in execution time. Moreover, our proposed OC has undergone rigorous testing, validating its effectiveness and efficiency in mitigating targeted multi-layered attacks. It has demonstrated precise detection capabilities against both application and data-link layer attacks. Regarding the limitations of our proposed OC, it runs on the Intel processor, offering SIMD architecture for parallel log data scanning. Therefore the OC provides enhanced and optimized results but with Intel processors, a point policymakers should be aware of.

In the future, more cyberattacks targeting multiple OSI layers can be tested with the OC. Additional features like cloud support and more performance metrics can be considered. Furthermore, OC can be utilized in other domains that involve extensive file scanning, such as SOAR systems, digital forensics, malware analysis, and data mining.

## Figures and Tables

**Table 1:** Correlation engines.

Correlation Engine	Development Platform	Memory Requirement	Platform Dependency	Usability
SEC [38]	Perl	Low	Independent	Easy
Esper [39]	Java and .Net	High	Both	Easy
Drools [40]	Java	High	Independent	Complex
NodeBrain [41]	C	Low	Dependent	Complex
Prelude [42]	Python	Low	Independent	Complex
OSSEC [43]	C	Low	Dependent	Easy
OSSIM [44]	C	Low	Dependent	Complex

**Table 2:** Feature comparison of our proposed OC and SEC.

Feature	SEC	Proposed OC
Engine type	Rule-base	Rule-base
Development environment	Perl	C
Application model	Single-threaded	Multi-threaded
Parallelism	No	Yes
Architecture requirement	No	SIMD
Rules processing	Sequential access	Parallel access
Data processing	Single data blocks	Multiple data block
Matching mechanism	Sequential regex matching	Parallel pattern matching
Core matching technique	Perl regex matching	Hyperscan

**Table 3:** System and VM details.

Feature	Setting/Value
Processor	Intel(R) Core(TM) i7-8550U
	CPU@1.80 GHz 2.00 GHz
RAM	8 GB
VM OS	CentOS 7
VM RAM	4 GB
No. of Processors (VM)	04

**Table 4:** Multi-layered attack detection.

Attack Type	Targeted OSI Layer	Detection Status
Simple GoldenEye DoS	Application	Detected
Random GoldenEye DoS	Application	Detected
Slow HTTP DoS	Application	Detected
SlowLoris DoS	Application	Detected
FTP	Application	Detected
Failed Login	Application	Detected
MAC Flooding	Data-Link	Detected
STP Root Take Over	Data-Link	Detected

## Data Availability

The original contributions presented in the study are included in the article; further inquiries can be directed to the corresponding authors.
